# Silent Bedrooms, Speaking Bodies: A Case Series on Hidden Emotional and Intimacy-Related Distress Behind Chronic Psychosomatic Symptoms in Women

**DOI:** 10.7759/cureus.110392

**Published:** 2026-06-07

**Authors:** Dhiraj Raja

**Affiliations:** 1 Psychiatry, Heritage Institute of Medical Sciences, Varanasi, IND

**Keywords:** couple counseling, emotional loneliness, emotional suppression, female mental health, intimacy-related distress, marital conflict, marital dissatisfaction, psychosexual distress, psychosomatic symptoms, somatic symptom disorder

## Abstract

Women frequently present with chronic bodily pain, gastritis, dizziness, fatigue, anxiety, panic symptoms, sleep disturbances, and other persistent physical complaints despite repeated investigations and treatment. Many consult multiple specialists, including physicians, neurologists, gastroenterologists, and gynecologists, before eventually reaching psychiatric services. In some women, hidden emotional distress, dissatisfaction within relationships, emotional loneliness, reduced emotional and physical intimacy, and unresolved psychosexual concerns may contribute significantly to persistent psychosomatic suffering.

We describe five married women from diverse sociocultural and religious backgrounds who continued to experience chronic physical symptoms despite repeated medical consultations and largely unremarkable investigations. Most patients initially focused solely on bodily complaints, including body pain, gastritis, dizziness, fatigue, panic symptoms, anxiety, and disturbed sleep. They found it difficult to openly discuss emotional stress, marital dissatisfaction, loneliness, or intimacy-related concerns because of shame, guilt, fear of conflict, dependency, and social pressure.

All patients underwent detailed psychiatric assessments, developmental and marital history-taking, supportive interviewing, and couple-based discussions led jointly by male and female psychiatrists. During extended clinical sessions, deeper emotional struggles gradually became clear. Several women described years of feeling emotionally neglected, unheard, disconnected, or dissatisfied in their relationships. Many also struggled to express emotional and sexual needs openly despite significant emotional suffering. In several cases, psychosomatic symptoms worsened during periods of loneliness, emotional conflict, rejection, or impaired communication within marriage.

We treated patients with supportive psychotherapy, emotional communication work, psychoeducation, stress-management techniques, couple counseling, sex education, and symptom-targeted medications. During follow-up visits, most patients reported improvement in psychosomatic symptoms, anxiety, sleep, emotional communication, marital functioning, and overall emotional well-being.

This case series explored whether hidden emotional and intimacy-related distress may represent possible contributing factors in chronic psychosomatic symptom experiences among some women within specific marital and sociocultural contexts.

## Introduction

Doctors frequently encounter women who continue to experience body pain, gastritis, fatigue, dizziness, sleep disturbances, headaches, panic symptoms, anxiety, and other physical complaints despite repeated investigations and treatment [1,2]. Many patients move from one medical specialty to another for years, while routine investigations remain largely normal. Despite multiple consultations and medications, their suffering often persists without significant relief.

Engel's biopsychosocial model explains that illness does not arise solely from biological disease [3]. Emotional distress, unresolved interpersonal conflict, relationship difficulties, trauma, and psychological suffering can also affect physical health and bodily functioning [4]. Long-term emotional stress may affect sleep, gastrointestinal symptoms, pain perception, autonomic functioning, emotional regulation, and overall quality of life.

While not universal, in some societies and conservative family settings, some women may find it difficult to openly express emotional or sexual dissatisfaction because of shame, fear of conflict, dependency, family expectations, or sociocultural pressure [5,6]. Some women may also hesitate to discuss marital dissatisfaction, impaired intimacy, emotional neglect, or psychosexual distress, even with close family members. Over time, unresolved emotional conflict may contribute to psychosomatic symptoms or be expressed through bodily symptoms rather than direct emotional communication.

International studies have shown significant associations among depression, anxiety, relationship dissatisfaction, female sexual dysfunction, and medically unexplained somatic symptoms [7,8]. Indian studies have also highlighted the role of emotional suppression, marital stress, gender expectations, and restricted emotional expression in women's mental health problems [9]. Freud's psychodynamic theories historically proposed that suppressed emotional and psychosexual conflicts may sometimes become expressed through physical symptoms when direct emotional expression becomes psychologically difficult [10]. Contemporary biopsychosocial, attachment-informed, trauma-informed, and interpersonal frameworks have further expanded this understanding by recognizing the complex interaction between emotional regulation, relational experiences, psychological stress, and bodily symptom expression.

Later psychological and psychosexual research also emphasized that emotional intimacy, relationship satisfaction, communication difficulties, guilt, shame, unresolved emotional needs, and psychosexual concerns may independently influence women's emotional well-being, relational functioning, and sexual health [11,12]. Many women with chronic psychosomatic symptoms may therefore silently carry emotional loneliness, marital dissatisfaction, impaired intimacy, or psychosexual frustration for years without openly discussing these experiences.

However, most psychosomatic literature continues to focus primarily on anxiety, depression, stress, or somatization, without thoroughly exploring hidden marital and intimacy-related conflicts during detailed psychiatric assessment [13]. In routine clinical practice, many women do not initially present with direct emotional or sexual complaints. Instead, they repeatedly seek treatment for physical symptoms such as gastritis, body pain, sleep disturbances, fatigue, headaches, panic attacks, dizziness, or anxiety.

We wrote this case series after repeatedly observing a similar hidden pattern among women presenting with chronic, unexplained physical symptoms. Behind years of psychosomatic suffering, we identified emotional loneliness, marital dissatisfaction, impaired emotional and physical intimacy, psychosexual frustration, difficulty openly communicating emotional needs, and long-standing suppression of emotional distress. We believe many women silently experience similar struggles, yet these experiences often remain under-recognized, under-discussed, and insufficiently explored during routine clinical assessment.

## Case presentation

Case series

Five married women presented to psychiatric services with persistent physical complaints despite repeated consultations and treatment across multiple medical specialties. Common symptoms included gastritis, generalized body pain, fatigue, dizziness, panic symptoms, anxiety, sleep disturbances, irritability, and excessive worry about physical health.

The patients came from diverse sociocultural and religious backgrounds commonly seen in North India, including Hindu, Muslim, and other religious families. We conducted comprehensive psychiatric assessments, developmental and marital history-taking, supportive interviewing, and couple-based discussions jointly through male and female psychiatrists. During extended clinical sessions, we explored emotional stress, relationship dynamics, personality patterns, intimacy-related concerns, and hidden emotional conflicts in detail. Using Diagnostic and Statistical Manual of Mental Disorders, 5th Edition (DSM-5)- and International Classification of Diseases, 11th Revision (ICD-11)-based clinical formulations, we assessed associated anxiety symptoms, depressive features, somatic symptom-related psychopathology, emotional dysregulation, and relational distress. Relevant medical investigations and specialist evaluations did not identify any major physical illness sufficient to fully explain the severity, persistence, and recurrence of symptoms.

We conducted all interviews and couple-based discussions sensitively within routine psychiatric and psychotherapeutic practice while maintaining professional boundaries, confidentiality, emotional safety, and voluntary participation throughout the clinical process.

Consent

Written informed consent was obtained from all patients for publication of this case series. We explained in detail the purpose of the publication, confidentiality measures, and the voluntary nature of participation. We conducted all psychiatric interviews and couple-based discussions sensitively within routine clinical practice while maintaining professional boundaries, emotional safety, and patient comfort throughout the assessment process. Patients retained the freedom to decline discussion of distressing emotional, marital, or intimacy-related concerns at any stage of evaluation or treatment. We removed or modified identifying personal and relational details wherever necessary to protect confidentiality and minimize the risk of identification.

Case 1

A 40-year-old married woman presented with recurrent complaints of gastritis, panic attacks, generalized body pain, sleep disturbance, palpitations, anxiety, and persistent fear about her health for nearly five years. Before psychiatric referral, she had consulted multiple physicians, neurologists, and gastroenterologists. Thyroid profile, imaging studies, and routine investigations remained within normal limits.

Initially, she focused solely on physical symptoms and repeatedly stated that doctors were “unable to identify the real disease.” She appeared emotionally guarded and avoided discussing marital or emotional concerns in detail. Overextended psychiatric discussions, deeper emotional distress gradually emerged.

She described feeling emotionally isolated in her marriage for many years. Communication with her husband had steadily declined, and intimacy had become emotionally distant and unfulfilling. She reported longing for affection, emotional closeness, and understanding, but avoided expressing these needs openly because of shame, guilt, and fear of conflict.

During further clinical exploration, she disclosed intense guilt about an emotionally close extramarital attachment that developed during a prolonged period of emotional neglect in her marriage. She described feeling trapped between emotional needs, social values, guilt, and marital responsibilities. She repeatedly noted that episodes of panic, gastritis, and bodily pain worsened after emotional stress or intimacy-related conflict.

During couple-based discussions, her husband acknowledged longstanding emotional withdrawal, occupational stress, unresolved grief over a previous relationship, and difficulty openly expressing emotions, despite caring for his wife. Overall assessment suggested that years of emotional neglect, marital dissatisfaction, impaired intimacy, unresolved guilt, and suppression of emotional and sexual needs had become closely intertwined with her psychosomatic symptoms.

We treated the couple with supportive psychotherapy, emotional communication work, psychoeducation, stress-management techniques, couple counseling, sex education, and escitalopram-clonazepam therapy. During follow-up visits, panic attacks decreased significantly, sleep improved, bodily pain lessened, and both partners gradually reported healthier emotional communication and greater intimacy.

Case 2

A 32-year-old married woman presented with dizziness, abdominal discomfort, panic-like episodes, fatigue, anxiety, sleep disturbance, and recurrent physical discomfort over nearly three years. She had already received treatment from multiple specialties, but relief was temporary. Most investigations were normal except for mild anemia.

She grew up in a conservative joint-family environment in which open discussion of emotions, relationships, or sexuality was strongly discouraged. During psychiatric assessment, she appeared shy, hesitant, and emotionally restricted. Initially, she reported only weakness, dizziness, and stomach discomfort, avoiding detailed discussion of her personal life.

During extended supportive discussions, she gradually described emotional dissatisfaction in her marriage. She stated that emotional closeness with her husband had declined significantly after childbirth. Although she continued to manage family responsibilities, she felt emotionally neglected and psychologically unsupported. She admitted that intimacy had become emotionally unsatisfying, but she never discussed these concerns openly because she feared rejection, blame, and family conflict.

Over time, suppressed emotional frustration appeared to manifest as anxiety, disturbed sleep, abdominal discomfort, dizziness, fatigue, and panic symptoms. Clinical assessment suggested longstanding suppression of emotional and intimacy-related dissatisfaction.

We treated her with supportive psychotherapy, emotional validation, psychoeducation, stress-management interventions, couple counseling, sex education, sertraline, and levosulpiride. During follow-up visits, her psychosomatic symptoms decreased significantly. She gradually became more emotionally expressive and reported improvements in communication, emotional closeness, and intimacy within the relationship.

Case 3

A 38-year-old married woman presented with recurrent gastric discomfort, generalized body pain, fatigue, sleep disturbances, anxiety symptoms, and low confidence, all persisting for nearly four years. Multiple medical evaluations, including gastrointestinal consultations, failed to identify any major physical illness that could adequately explain the severity of her symptoms.

A detailed psychiatric assessment revealed longstanding body-image insecurity related to a congenital deformity of the left index finger. She described repeated experiences of rejection before marriage, which gradually eroded her confidence, self-worth, femininity, and emotional expression.

Initially, she described her marriage as “normal.” However, extended clinical discussions revealed emotional distance, dissatisfaction with intimacy, and a sense of emotional disconnection from her husband. She admitted to avoiding open discussion of dissatisfaction with intimacy because of embarrassment, fear of rejection, and low self-esteem. Emotional insecurity, dissatisfaction within intimacy, suppressed emotional needs, and low self-worth appeared closely associated with worsening body pain, fatigue, gastric symptoms, and sleep disturbances.

We treated her with supportive psychotherapy, self-esteem-focused counseling, psychoeducation, emotional expression work, couple counseling, escitalopram-clonazepam therapy, and rabeprazole-levosulpiride treatment. During follow-up visits, she gradually reported increased confidence, improved sleep, less body pain, and healthier emotional communication within the relationship.

Case 4

A 32-year-old married woman presented with chronic gastritis, panic symptoms, dizziness, anxiety, irritability, sleep disturbance, and multiple medically unexplained physical complaints that had persisted for nearly four years despite repeated treatment across multiple specialties. Detailed developmental history revealed early paternal loss, followed by emotional insecurity, teasing, chronic fearfulness, and inadequate emotional support during adolescence. After marriage, prolonged separation from her husband for occupational reasons further intensified emotional loneliness and dissatisfaction.

She repeatedly described feeling emotionally unsupported and psychologically isolated for years. She admitted difficulty openly discussing emotional and intimacy-related dissatisfaction because she feared being perceived as “demanding” or “selfish.” Over time, emotional deprivation appeared closely associated with panic symptoms, gastritis, dizziness, irritability, sleep disturbance, and generalized psychosomatic distress and may have contributed to worsening symptom experiences during periods of emotional conflict. Clinical assessment suggested longstanding suppression of emotional loneliness, unresolved fear, inadequate emotional support, and marital dissatisfaction.

We treated her with supportive psychotherapy, trauma-focused emotional work, stress-management interventions, couple counseling, fluoxetine, clonazepam, and low-dose olanzapine. During follow-up visits, anxiety decreased significantly, sleep improved, psychosomatic symptoms lessened, and emotional communication within the marriage gradually improved.

Case 5

A 25-year-old married woman was referred from neurology with depressive symptoms, generalized body pain, gastritis, anxiety, irritability, and disturbed sleep that began around six months after childbirth. She had received treatment from multiple specialties for nearly two years with minimal improvement. Thyroid profile, NCCT of the head, and routine investigations remained normal.

During psychiatric assessment, she repeatedly described feeling emotionally lonely after childbirth due to a prolonged separation from her husband for occupational reasons. She stated that emotional and physical intimacy had declined significantly after delivery and during the separation. Despite significant emotional distress, she avoided openly discussing dissatisfaction because of fear of conflict, judgment, and misunderstanding within the family.

She repeatedly described feeling emotionally abandoned despite remaining socially married. Over time, unresolved loneliness, emotional deprivation, reduced intimacy, and dissatisfaction within the relationship gradually manifested as depressive symptoms, body pain, gastritis, disturbed sleep, irritability, and anxiety.

Overall clinical assessment suggested that suppressed emotional needs, chronic emotional disconnection, and dissatisfaction with intimacy may have been associated with her psychosomatic symptom experiences and may have contributed to the persistence or worsening of emotional distress during periods of relational conflict.

We treated her with supportive psychotherapy, psychoeducation, emotional communication work, stress-management interventions, and escitalopram therapy. During follow-up visits, she reported improvements in mood, sleep, body pain, emotional functioning, and marital communication. These improvements remained stable throughout follow-up.

What ties these cases together?

Although these women came from diverse personal, sociocultural, and religious backgrounds, all five cases revealed a strikingly similar emotional pattern underlying the physical symptoms. None of the patients initially presented with direct complaints about intimacy, emotional dissatisfaction, or marital conflict. Instead, they primarily sought treatment for chronic physical symptoms, including gastritis, body pain, fatigue, dizziness, panic symptoms, anxiety, and disturbed sleep.

Detailed psychiatric assessments revealed longstanding emotional loneliness, marital dissatisfaction, reduced emotional and physical intimacy, difficulty openly expressing emotional and sexual needs, and chronic suppression of emotional distress across cases. Many women silently carried feelings of emotional neglect, guilt, frustration, emotional disconnection, or unmet emotional needs for years while continuing to fulfill family and social responsibilities.

Another important observation was the close relationship between emotional conflict and symptom severity. Many patients reported worsening physical symptoms during periods of loneliness, rejection, separation, emotional conflict, or impaired communication within relationships. Improvement became more noticeable after emotional validation, supportive psychotherapy, couple-based discussions, and the restoration of healthier emotional communication.

Taken together, these cases suggest that hidden emotional and intimacy-related distress was explored as a possible contributing factor in these psychosomatic symptom experiences among women who felt unable to communicate emotional suffering within their marital or sociocultural environment openly (Table 1).

**Table 1:** A summary of the clinical characteristics, hidden emotional and psychosexual stressors, treatment, and outcomes in five women with chronic psychosomatic symptoms All five women presented with chronic psychosomatic symptoms despite repeated consultations and treatment across multiple medical specialties. Common complaints included gastritis, generalized body pain, fatigue, dizziness, panic symptoms, anxiety, sleep disturbance, irritability, depressive symptoms, and excessive concern about physical health. Most patients had already undergone repeated medical investigations before psychiatric referral, including gastrointestinal evaluation, neurological assessment, thyroid profile, imaging studies, and routine laboratory tests, none of which revealed major organic pathology sufficient to explain the severity and persistence of symptoms. All patients underwent a detailed psychiatric assessment using DSM-5- and ICD-11-based clinical evaluation. We conducted developmental and marital history-taking, supportive interviewing, and couple-based discussions, with male and female psychiatrists working jointly. During extended clinical sessions, we explored emotional stress, relationship dynamics, emotional communication, intimacy-related concerns, and hidden emotional conflicts in depth. Most women initially focused only on bodily complaints. They hesitated to discuss emotional suffering, marital dissatisfaction, loneliness, or intimacy-related concerns because of shame, fear of conflict, guilt, and sociocultural pressure. Across cases, we observed recurring patterns of emotional loneliness, reduced emotional and physical intimacy, emotional neglect, impaired communication, suppression of emotional and sexual needs, and unresolved emotional distress. Several patients reported worsening psychosomatic symptoms during periods of emotional conflict, rejection, loneliness, separation, or impaired marital communication. Most patients gradually improved with supportive psychotherapy, psychoeducation, emotional communication work, couple counseling, stress-management interventions, sex education, and symptom-based pharmacological treatment

Case	Age (years)	Duration of symptoms	Main symptoms	Major emotional and psychosexual stressors	Treatment/medication	Outcome
1	40	~5 years	Gastritis, panic symptoms, body pain, disturbed sleep, anxiety	Emotional neglect, marital dissatisfaction, reduced intimacy, unresolved guilt, and emotional suppression	Supportive psychotherapy, couple counselling, psychoeducation, and escitalopram-clonazepam	Anxiety, sleep, body pain, and emotional communication improved during follow-up
2	32	~3 years	Panic symptoms, dizziness, abdominal discomfort, disturbed sleep	Financial stress, emotional suppression, dissatisfaction within marriage, and reduced intimacy	Supportive psychotherapy, couple counselling, sertraline, and levosulpiride	Marked improvement in anxiety, sleep, psychosomatic symptoms, and marital functioning
3	38	~4 years	Gastric discomfort, fatigue, body aches, disturbed sleep	Body-image insecurity, emotional inhibition, dissatisfaction within intimacy, and marital emotional distance	Supportive psychotherapy, self-esteem counselling, escitalopram-clonazepam, and rabeprazole-levosulpiride	Body pain, confidence, sleep, and emotional communication gradually improved
4	32	~4 years	Gastric complaints, panic symptoms, dizziness, disturbed sleep	Childhood trauma, emotional neglect, prolonged emotional loneliness, and marital stress	Supportive psychotherapy, couple counselling, fluoxetine, clonazepam, and low-dose olanzapine	Anxiety, sleep, emotional functioning, and physical symptoms improved
5	25	~2 years	Body pain, gastritis, depressive symptoms, anxiety	Emotional loneliness, prolonged marital separation, reduced intimacy, and suppressed emotional needs	Supportive psychotherapy, psychoeducation, escitalopram, levosulpiride, and pregabalin-methylcobalamin	Significant improvement with sustained remission during follow-up

## Discussion

Among all five patients, emotional distress appeared closely associated with worsening physical symptoms. None of the women initially presented with direct complaints about emotional dissatisfaction, intimacy, or marital conflict. Instead, most women primarily described gastritis, body pain, fatigue, dizziness, panic symptoms, anxiety, disturbed sleep, or excessive worry about physical health. Several had already spent years consulting various specialists before seeking psychiatric services.

During detailed psychiatric assessments and extended clinical discussions, deeper emotional struggles gradually emerged. Many women had suppressed emotional distress for years because of shame, fear of judgment, social expectations, dependency, or difficulty discussing emotional and intimacy-related concerns openly. In several cases, physical symptoms appeared to worsen during periods of emotional distress, relational conflict, loneliness, or impaired communication.

Across all cases, a striking observation involved hidden dissatisfaction within marital relationships. Several women described emotional loneliness despite remaining socially married. Some reported years of feeling emotionally neglected, psychologically disconnected, unheard, or physically distant from their partners, while a few described intimacy as emotionally unfulfilling. Despite significant distress, many avoided openly discussing these experiences because of guilt, fear of conflict, rejection, misunderstanding, or family consequences.

In many patients, psychosomatic symptoms worsened during periods of emotional conflict, loneliness, rejection, separation, or impaired communication within relationships. This pattern repeatedly emerged during detailed clinical assessments and follow-up visits. Emotional distress, dissatisfaction within intimacy, chronic stress, relational difficulties, anxiety symptoms, depressive features, and suppression of emotional needs appeared closely intertwined with worsening psychosomatic symptom experiences across cases.

Previous Indian studies primarily focused on anxiety, depression, somatization, sociocultural stress, and gender-related pressures affecting women’s mental health [5,9,12]. Similarly, much of the international literature treated female sexual dysfunction, relationship dissatisfaction, depression, and medically unexplained symptoms as largely separate clinical entities [7,8,11,13]. In contrast, our case series explored whether hidden emotional loneliness, impaired emotional and physical intimacy, relational distress, and suppressed emotional conflict may represent possible contributing factors in psychosomatic symptom experiences among some women within specific marital and sociocultural contexts.

This observation has important clinical relevance because many women do not initially disclose emotional or intimacy-related dissatisfaction during routine consultations. Instead, they repeatedly seek treatment for body pain, gastritis, fatigue, sleep disturbances, dizziness, headaches, panic symptoms, or anxiety. Unless clinicians create a psychologically safe environment and sensitively explore relationship experiences in depth, these hidden emotional struggles may remain clinically invisible.

Another important observation was that psychosexual dissatisfaction rarely occurred in isolation. Most women also experienced emotional neglect, loneliness, poor communication, unresolved guilt, body-image insecurity, emotional suppression, trauma-related distress, or sociocultural restrictions on emotional expression. These factors appeared deeply interconnected rather than as separate psychological problems.

Improvement did not occur with medications alone. Several patients showed more meaningful improvement after emotional validation, supportive psychotherapy, emotional reassurance, psychoeducation, couple-based discussions, and healthier emotional communication within relationships. As emotional expression gradually improved, psychosomatic symptoms also decreased in many patients.

We intentionally wrote this case series in a clinically direct and emotionally honest manner because many women silently continue to suffer from chronic physical symptoms that are often repeatedly medicalized yet insufficiently explored emotionally. Our intention was not to reduce psychosomatic symptoms to sexuality or marital dissatisfaction but to highlight an important emotional reality that may remain hidden during routine clinical practice.

What is unique about our case series?

Unlike many earlier Indian and international studies that examined anxiety, depression, somatization, stress, or female sexual dysfunction in isolation, our case series explored how hidden emotional loneliness, marital dissatisfaction, impaired emotional and physical intimacy, and suppressed psychosexual distress may silently manifest as chronic psychosomatic symptoms in women. Another important difference was that none of the patients initially presented with direct emotional or sexual complaints. Most women primarily sought treatment for physical symptoms such as gastritis, body pain, fatigue, dizziness, panic symptoms, anxiety, and disturbed sleep. Detailed psychiatric assessment and couple-based discussions later revealed deeper emotional and intimacy-related conflicts that had remained suppressed for years. Our series also emphasized real-world clinical exploration rather than a purely questionnaire-based approach, highlighting an under-recognized emotional reality commonly encountered in routine psychiatric practice.

Clinical insight

This case series highlights an important clinical reality that clinicians may overlook during routine consultations. Women experiencing hidden emotional loneliness, marital dissatisfaction, impaired emotional and physical intimacy, and suppressed psychosexual distress may initially present with chronic physical complaints rather than direct emotional or sexual concerns. Symptoms such as gastritis, body pain, fatigue, dizziness, panic symptoms, disturbed sleep, and anxiety may therefore reflect deeper, unresolved emotional suffering in some patients. Clinicians may improve identification and treatment outcomes by creating an emotionally safe clinical environment and sensitively exploring relationship dynamics, emotional stress, and intimacy-related concerns during psychiatric assessment.

Limitations

This case series has several limitations. First, the observational design limits our ability to establish direct causal relationships between emotional distress, relational difficulties, and psychosomatic symptoms. Second, our psychological and psychodynamic interpretations primarily emerged from detailed clinical interviews, longitudinal psychiatric observation, and patient disclosures during assessment and follow-up rather than from structured research-based assessment models. Third, we did not systematically use standardized psychometric instruments to assess anxiety, depression, somatic symptom severity, trauma-related distress, marital satisfaction, attachment-related patterns, or sexual functioning because we primarily followed a clinical, exploratory, and interview-based approach. The absence of validated psychometric measures limits the reliability, reproducibility, objective quantification, and interpretive rigor of the findings. Fourth, the small sample size limits broader generalizability. Finally, sociocultural sensitivity regarding emotional and intimacy-related concerns may have influenced the depth, timing, and extent of emotional disclosure during interviews. Future studies should incorporate validated psychometric scales, structured longitudinal assessment, and larger samples to evaluate better the complex relationship between psychosomatic symptoms, emotional distress, relational functioning, and psychosexual well-being.

## Conclusions

This exploratory case series observed recurring patterns of emotional loneliness, marital dissatisfaction, impaired emotional and physical intimacy, emotional suppression, and relational distress among women presenting with chronic psychosomatic symptoms. However, the observational design, small sample size, and absence of standardized psychometric assessment do not permit causal conclusions regarding these associations. Many patients initially presented with persistent physical complaints while deeper emotional distress remained unspoken or clinically underexplored. Careful psychiatric assessment and sensitive exploration of emotional, relational, and psychosocial experiences may help clinicians better understand the multidimensional nature of psychosomatic symptom presentations in a safe, supportive clinical environment.
